# Joint models quantify associations between immune cell kinetics and allo-immunological events after allogeneic stem cell transplantation and subsequent donor lymphocyte infusion

**DOI:** 10.3389/fimmu.2023.1208814

**Published:** 2023-08-01

**Authors:** Eva A. S. Koster, Edouard F. Bonneville, Peter A. von dem Borne, Peter van Balen, Erik W. A. Marijt, Jennifer M. L. Tjon, Tjeerd J. F. Snijders, Daniëlle van Lammeren, Hendrik Veelken, Hein Putter, J. H. Frederik Falkenburg, Constantijn J. M. Halkes, Liesbeth C. de Wreede

**Affiliations:** ^1^ Department of Hematology, Leiden University Medical Center, Leiden, Netherlands; ^2^ Department of Biomedical Data Sciences, Leiden University Medical Center, Leiden, Netherlands; ^3^ Department of Hematology, Medisch Spectrum Twente, Enschede, Netherlands; ^4^ Department of Hematology, HagaZiekenhuis, The Hague, Netherlands

**Keywords:** T-cell kinetics, joint modelling, allogeneic stem cell transplantation, donor lymphocyte infusion, graft-versus-host-disease, T-cell depletion, acute myeloid leukemia, acute lymphoblastic leukemia

## Abstract

Alloreactive donor-derived T-cells play a pivotal role in alloimmune responses after allogeneic hematopoietic stem cell transplantation (alloSCT); both in the relapse-preventing Graft-versus-Leukemia (GvL) effect and the potentially lethal complication Graft-versus-Host-Disease (GvHD). The balance between GvL and GvHD can be shifted by removing T-cells via T-cell depletion (TCD) to reduce the risk of GvHD, and by introducing additional donor T-cells (donor lymphocyte infusions [DLI]) to boost the GvL effect. However, the association between T-cell kinetics and the occurrence of allo-immunological events has not been clearly demonstrated yet. Therefore, we investigated the complex associations between the T-cell kinetics and alloimmune responses in a cohort of 166 acute leukemia patients receiving alemtuzumab-based TCD alloSCT. Of these patients, 62 with an anticipated high risk of relapse were scheduled to receive a prophylactic DLI at 3 months after transplant. In this setting, we applied joint modelling which allowed us to better capture the complex interplay between DLI, T-cell kinetics, GvHD and relapse than traditional statistical methods. We demonstrate that DLI can induce detectable T-cell expansion, leading to an increase in total, CD4+ and CD8+ T-cell counts starting at 3 months after alloSCT. CD4+ T-cells showed the strongest association with the development of alloimmune responses: higher CD4 counts increased the risk of GvHD (hazard ratio 2.44, 95% confidence interval 1.45-4.12) and decreased the risk of relapse (hazard ratio 0.65, 95% confidence interval 0.45-0.92). Similar models showed that natural killer cells recovered rapidly after alloSCT and were associated with a lower risk of relapse (HR 0.62, 95%-CI 0.41-0.93). The results of this study advocate the use of joint models to further study immune cell kinetics in different settings.

## 1 Introduction

The curative potential of allogeneic stem cell transplantation (alloSCT) in the treatment of hematological malignancies depends on the introduction of donor-derived alloreactive T-cells (1). These T-cells recognize non-self antigens on patient-derived cells and can, once activated, expand and eliminate those cells. Targeting antigens on lymphohematopoietic cells including the malignant cells leads to the desired Graft-versus-Leukemia (GvL) effect and prevents relapse. However, when other tissues of the patient are targeted, Graft-versus-Host-Disease (GvHD) may develop (2). Natural killer (NK) cells may discriminate between healthy and non-healthy (e.g., virus-infected or malignant) cells by acting on signals from inhibitory and activating receptors that bind to the target cell. In the setting of alloSCT, early NK cell recovery can protect against relapse and viral infections (3, 4). However, NK cells do not appear to be important effector cells in GvHD (5).

To reduce the risk of severe GvHD, donor T-cell depletion (TCD) can be applied, although this will decrease the GvL effect (6). In order to restore the GvL effect to prevent relapse, TCD alloSCT can be combined with the administration of donor lymphocyte infusions (DLIs) after transplant (2, 7, 8). DLI as part of a pre-emptive strategy is administered to patients with detectable minimal residual disease (MRD) or with residual patient hematopoiesis: mixed chimerism (MC). DLI as part of a prophylactic strategy is given to all patients in whom no GvHD has developed as sign of alloreactivity. The alloreactive potential of DLI decreases over time after alloSCT: both the efficacy (GvL effect) and toxicity (GvHD) are highest early after alloSCT (9, 10). Therefore, administration preferably starts a few months after alloSCT to allow for sufficient GvL without severe GvHD (11).

Since T-cells are pivotal for alloimmune responses, several groups have investigated T-cell kinetics after alloSCT and their impact on the development of GvHD or relapse. However, as shown in the recent review by Yanir et al. (12), the reported results are inconsistent, and their interpretation is complicated by several factors. First, T-cells can be patient- or donor-derived, while only donor-derived T-cells are responsible for GvHD and GvL. Second, the T-cell changes following alloSCT are the combined result of *de novo* T-cell generation from infused hematopoietic stem cells starting at least 6 months after alloSCT, homeostatic proliferation of T-cells present in the patient or graft, T-cell expansion during infections and expansion of alloreactive T-cells responsible for GvL and GvHD. Especially cytomegalovirus (CMV) reactivations are common during the first 3 months after alloSCT and strongly affect the kinetics of both T-cells and NK cells after alloSCT (13–15). This may distort the association between the kinetics of the main T-cell subsets and specific alloimmune responses, i.e., the presence of GvHD and the absence of relapse as a result of the GvL effect. Third, factors that could influence both the T-cell kinetics and the risks of GvHD and relapse, such as the conditioning regimen, donor type and the use and method of TCD, should be properly accounted for. Finally, ignoring clinical events or interventions during follow-up can also be problematic: over time, the patients that have not yet experienced an event like relapse, death or the development of GvHD, become less representative of the population at the beginning of follow-up. As death by definition prevents further T-cell measurements and the possibility of experiencing subsequent GvHD and relapse, bias is created by considering the patients who died as having non-informatively dropped out (i.e. that their measurements *could* have been measured if kept under follow-up). Likewise, DLI and the use of posttransplant prophylactic immunosuppression are known to affect the risks of relapse and GvHD, but may also affect the T-cell kinetics (16–23). To fully understand the complex interplay between all these factors, sophisticated statistical methods are required that properly model the T-cell kinetics themselves, along with their association with GvHD or relapse. Joint modelling captures the T-cell trajectories and the clinical events simultaneously, accounting for informative dropout, as well as the measurement error and heterogeneity in individual trajectories (24).

In this study, we performed joint modelling to investigate the complex associations between the immune cell kinetics and alloreactivity in a cohort of 166 patients receiving an alloSCT for acute leukemia or myelodysplastic syndrome (MDS). All patients received an alemtuzumab-based TCD alloSCT after nonmyeloablative conditioning without any posttransplant prophylactic immunosuppression. Patients with an anticipated high risk of relapse were scheduled to receive an early low-dose DLI prophylactically at 3 months after alloSCT, while prophylactic DLI administration for the other patients started at 6 months. In this unique setting we investigated the impact of the early low-dose DLI on the T-cell and NK cell kinetics during the first 6 months after transplant and the association between these kinetics and the development of clinical events.

## 2 Methods

### 2.1 Study population

This retrospective study included all adult patients with acute myeloid leukemia, acute lymphoblastic leukemia or MDS in complete morphologic remission after intensive induction therapy who received their first alloSCT from a 9 or 10 out of 10 HLA matched donor using nonmyeloablative conditioning and alemtuzumab-based TCD (25) between March 2008 and December 2019 at Leiden University Medical Center (LUMC, Leiden, The Netherlands). Two patients who were transplanted while receiving systemic immunosuppression for a non-transplant indication (polymyalgia rheumatica and cryptogenic organizing pneumonia) were excluded because of the potential impact of the ongoing systemic immunosuppression on the immune cell recovery after alloSCT. All patients signed informed consent for data collection and analysis. Data were analyzed as of July 2021.

### 2.2 Transplantation and DLI strategy

As conditioning regimen patients received either fludarabine (6 days 50 mg/m^2^ orally or 30 mg/m^2^ intravenously) and busulfan (2 days 4x0.8 mg/kg intravenously), or the FLAMSA regimen: fludarabine (5 days 30 mg/m^2^ intravenously), cytarabine (4 days 2000 mg/m^2^ intravenously), amsacrine (4 days 100 mg/m^2^ intravenously) and busulfan (4 days 4x0.8 mg/kg intravenously). In both regimens, TCD was performed by adding 20 mg alemtuzumab (Sanofi Genzyme, Naarden, The Netherlands) to the graft before infusion and by administering 15 mg alemtuzumab intravenously on days -4 and -3. Patients with an unrelated donor (UD) received rabbit-derived anti-thymocyte globulin (ATG; Sanofi Genzyme) additionally on day -2 (until April 2010 2mg/kg and thereafter 1mg/kg). None of the patients received posttransplant GvHD prophylaxis.

The dose of unmodified pre-emptive and prophylactic DLIs was based on donor type and timing after alloSCT. Standard DLIs given at 6 months after alloSCT contained 3x10^6^ or 1.5x10^6^ T-cells/kg for patients with a related donor (RD) or an UD, respectively. Early low-dose DLIs given at 3 months after alloSCT contained 0.3x10^6^ or 0.15x10^6^ T-cells/kg for patients with a RD or an UD, respectively. Since May 2010, all patients without any relapse and without GvHD requiring systemic immunosuppressive treatment at 6 months after alloSCT prophylactically (i.e., irrespective of chimerism or posttransplant MRD status) were planned to receive the standard DLI. Patients who were considered to have a high risk of relapse based on the disease characteristics or MRD status at time of alloSCT or who received the FLAMSA regimen were also scheduled to receive the early low-dose DLI prophylactically at 3 months after alloSCT. All patients, including those transplanted before May 2010, could receive pre-emptive DLIs in case of MC or MRD positivity, starting from 3 months after alloSCT. Additionally, as part of several clinical trials, patients could receive modified T-cell products prophylactically or virus-specific T-cell infusions to treat severe viral infections.

### 2.3 Monitoring of CMV and absolute numbers of circulating immune cells

CMV serostatus was assessed in all patients and donors before alloSCT. After transplant CMV was monitored routinely by PCR on peripheral blood samples in all patients. Absolute numbers of circulating total (CD3+), CD4+CD8- and CD4-CD8+ T-cells, B cells and NK cells were measured routinely at predefined timepoints on anticoagulated fresh venous blood by flow cytometry with bead calibration (Trucount tubes, BD Biosciences). Samples were measured either on a FACSCalibur using anti-CD3-APC, anti-CD4-FITC, anti-CD8-PE, and anti-CD45-PerCP or with anti-CD3-FITC, anti-CD16-PE, anti-CD19-APC, anti-CD45-PerCP, and anti-CD56-PE, or on a FACSCanto using anti-CD3-APC, anti-CD4-PB, anti-CD8-FITC, anti-CD16-PE, anti-CD19-PE Cy7, anti-CD45-PerCP, and anti-CD56-PE (all from BD). The lower detection limit was 0.5x10^6^ cells/L.

### 2.4 Definitions of events

Relapse was defined as the recurrence of at least 5% blasts on cytomorphologic bone marrow examination or at least 1% blasts in peripheral blood (if possible, confirmed by BM biopsy). We defined clinically significant GvHD as the start of therapeutic systemic immunosuppression for GvHD (26). We defined ‘other failure’ as the occurrence of an adverse event with a potential impact on the immune cell kinetics: death, graft failure, start of systemic immunosuppression for a non-GvHD indication, and virus-specific T-cell infusion for a severe viral infection (whichever occurred first). Graft failure was defined as the occurrence of >95% patient BM chimerism in all lineages tested or refractory granulopenia (granulocyte count <0.5x10^9^/l) in the absence of relapse or ongoing myelotoxic medication.

For this study we analyzed the T-cell and NK cell kinetics and events during the first 6 months after alloSCT, during which the early immunological recovery and most CMV reactivations take place. Furthermore, during this period the impact of the early low-dose DLI can be assessed, as the standard DLI is given to all eligible patients around 6 months after alloSCT. As part of the analyses assessing the net impact of the early low-dose DLI on the T-cell and NK cell kinetics and clinical events, patients receiving a standard DLI or modified T-cell product as part of a clinical trial were censored at 7 days after this infusion. We considered this to be non-informative censoring, since these interventions were prophylactic and not driven by the clinical course of the patient. For the T-cell kinetics we considered the circulating cell counts of the total (CD3+) T-cell population and the two major T-cell subpopulations: the CD4+CD8- and the CD4-CD8+ T-cells.

### 2.5 Statistical analyses

Probabilities of overall survival (OS) and relapse-free survival (RFS) after alloSCT with associated 95% confidence intervals (95%-CI) were calculated by the Kaplan-Meier method. The cumulative incidences of clinically significant GvHD and relapse from time of alloSCT were estimated by means of the Aalen-Johansen method, treating other failure (as described in the previous section) as a third competing risk.

To study the complex interplay between the immune cell kinetics, DLI and clinically relevant endpoints (GvHD and relapse), two joint models were developed; model I starting at time of alloSCT and model II at time of the early low-dose DLI.

Shared-parameter joint models consist of two components: a longitudinal submodel, and a time-to-event submodel (24). The former often takes the form of a mixed-effects regression model, and the latter is generally assumed to follow a proportional hazards structure, similar to a Cox model (for one or possibly multiple endpoints such as GvHD or relapse). The mixed-effects model allows to model cell count trajectories over time, while appropriately accounting for both the heterogeneity in subject-specific trajectories (using random effects) and measurement error. These two submodels are linked together via an association structure. Practically speaking, this allows the hazard of a particular event to depend on characteristics of an individual’s specific trajectory, such as the ‘true’ underlying (i.e. in absence of measurement error) value over time. In turn, this enables the estimation of an association between a longitudinal marker (e.g. CD3 counts) and the risk of a clinical event (e.g. GvHD). In the presence of an association, the estimated trajectories themselves will be corrected for bias related to the measurements being terminated by the occurrence of endpoints (generally known as ‘informative dropout’).

Below follows a concise description of the joint models developed for the present application. Detailed explanation of the statistical models and the underlying rationale can be found in the **Statistical Supplement**. For all models, absolute cell counts were analyzed on the log scale after setting measurements under the detection limit to 0.5. This only occurred at earliest timepoints where because of the lymphodepletion by the conditioning regimen and TCD, the counts are expected to be around zero.

#### 2.5.1 Model I (starting from alloSCT)

To investigate the effect of early low-dose DLI on the kinetics of the T-cell and NK cell counts after TCD alloSCT, we performed an intention-to-treat (ITT) analysis with a baseline group distinguishing between those scheduled for early low-dose DLI because of a high anticipated risk of relapse (henceforth ‘high risk’ group) and those who were not (‘non-high risk’ group). We chose this approach instead of a per-protocol analysis since we could not properly define a control group of patients who did not receive early DLI but could have been candidates as we did not know for each patient who was not scheduled for early DLI whether he/she would have been able to receive it.

**Figure 1:**
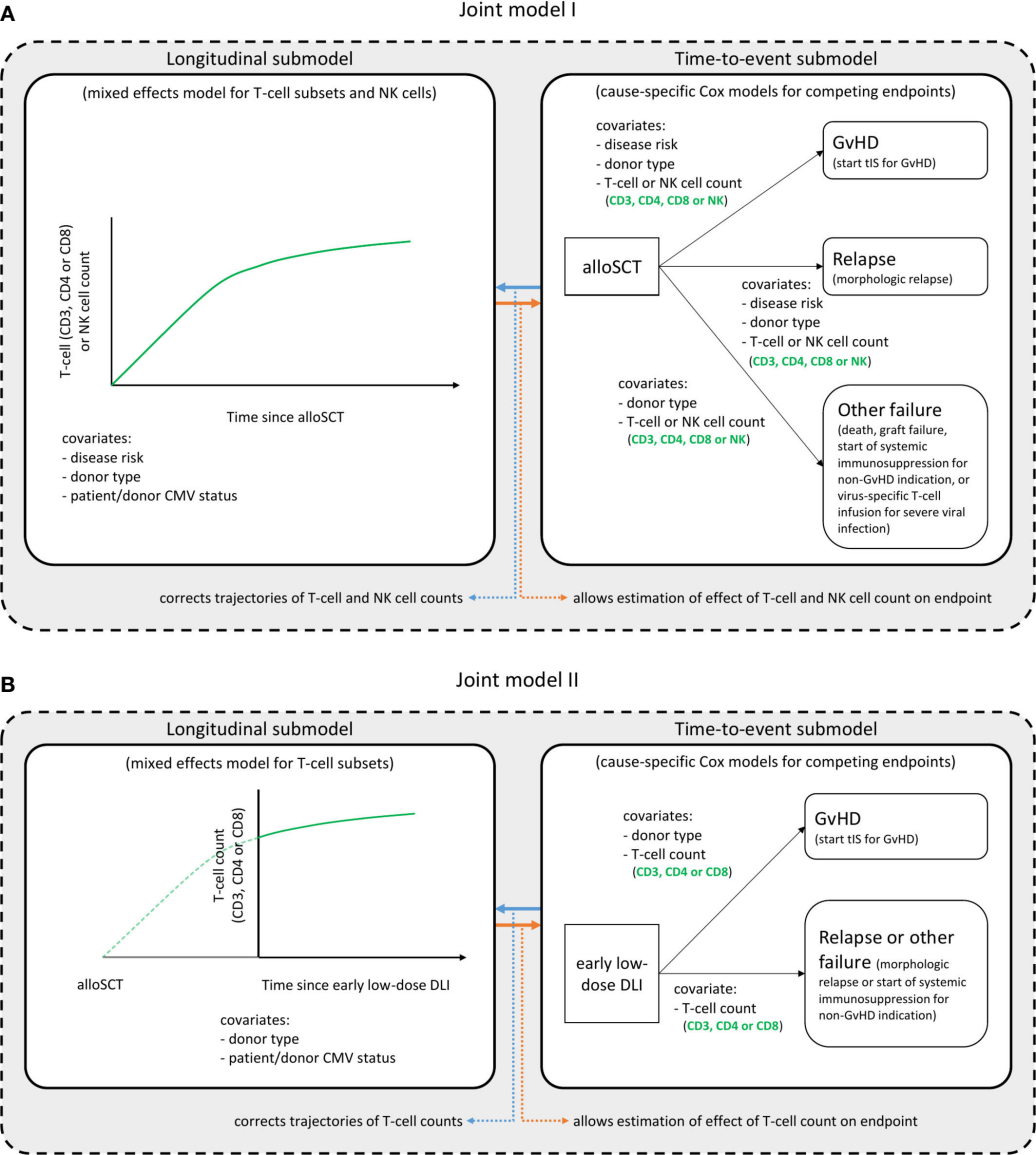
Structure of the joint models. Graphical description of the two joint models. Joint model I **(A)** starts at time of alloSCT, joint model II **(B)** at time of the early low-dose DLI. Each model consists of a longitudinal and a time-to-event submodel and was run in turn for each T-cell subset, considering either the CD3+, CD4+ or CD8+ T-cell counts, and the NK cell counts. These are the outcome of the longitudinal submodel and a time-dependent covariate in the time-to-event submodel. All other variables in each submodel are baseline covariates. Per endpoint of the time-to-event submodels, the clinical events that occurred during the relevant time period (first 6 months after alloSCT or first 3 months after the early low-dose DLI) are described. The NK cells were only analyzed in model I. See the Statistical Supplement for a detailed description of the model structures.

**Figure 1A** shows a schematic overview of joint model I. The model was run separately for each T-cell subset, respectively using CD3, CD4 or CD8 counts, and the total NK counts. All patients started at time of alloSCT and were followed-up until 6 months after alloSCT or until the occurrence of an earlier endpoint (GvHD, relapse or other failure), whichever occurred first. The longitudinal submodel was a linear mixed-effects model, which used restricted cubic splines to flexibly model the log counts over time. The baseline covariates included in this submodel were disease risk (non-high risk or high risk), donor type (RD or UD with ATG-containing conditioning regimen) and patient/donor CMV status (both seronegative [CMV -/-] or not). The patient/donor CMV status was included as simple fixed effect, and both disease risk and donor type were included as part of a three-way interaction with time. This was in order to both properly accommodate the expected slower lymphocyte recovery in patients treated with ATG, and to evaluate a difference in trajectories between the disease risk groups. The time-to-event submodel comprised three cause-specific proportional hazards models, with GvHD, relapse and other failure as competing events. As predictors, they each contained the time-dependent current value (i.e. the underlying ‘true’ value at a given timepoint, as estimated by the longitudinal submodel) of the log immune cell count, as well as the baseline factors donor type and disease risk. The latter was omitted as a covariate from the model for ‘other failure’ due to the limited number of events.

To investigate whether the current slope (i.e. rate of increase or decrease of counts at a given moment) of the T-cell counts was associated with the development of GvHD, we also extended the models by adding the current slope of the log counts in addition to the current value to the time-to-event submodel (so-called ‘time-dependent slopes’ parametrization).

#### 2.5.2 Model II (starting from early low-dose DLI)

To further investigate the T-cell kinetics after the early low-dose DLI, we constructed a joint model including only the patients who actually received the early low-dose DLI without any prior event of interest (**Figure 1B**). Since NK cells recover rapidly after alloSCT (27) (expected before the administration of early low-dose DLI in this study), they were not considered for model II. The time-scale was taken from DLI instead of from alloSCT, and follow-up was restricted to 3 months after this DLI, until administration of a second DLI, or until the occurrence of a terminating event, whichever occurred first. The disease risk factor was omitted since all included patients belonged to the high risk group. Since only 7 patients had a non-GvHD event within 3 months after the early low-dose DLI (**Supplementary Figure 1**), relapse and other failure were combined into one composite endpoint to compete with GvHD and the donor type factor was omitted for this composite endpoint.

### 2.6 Software

All analyses were performed in R version 4.2.1 using the packages JM (28) (version 1.5-2), survival (29) (version 3.4.0) and nlme (30) (3.1-157). Full code needed to reproduce the results of the present work is available at https://github.com/survival-lumc/ImmuneReconstJM, and structured using the targets (31) (version 0.14.0) package.

## 3 Results

### 3.1 Population

166 patients were included in this study. Baseline characteristics are presented in **Table 1**. All surviving patients had at least 12 months follow-up since alloSCT. OS and RFS at 6 months after alloSCT were 77% (95%-CI 71-83) and 70% (95%-CI 64-77), respectively. A total of 62 patients were considered to have a high risk of relapse and were scheduled for an early low-dose DLI, of whom 42 actually received it after a median interval of 3.1 months (range: 2.7-4.4) without any prior event of interest (**Supplementary Figure 1**). Twenty patients did not receive an early low-dose DLI: 10 because of early relapse, 9 because of early other failures (death [n=1], graft failure [n=2], start of systemic immunosuppression for a non-GvHD indication [n=4], or administration of a virus-specific T-cell infusion [n=2]), and 1 patient did not receive the early low-dose DLI because of mild skin GvHD requiring topical treatment. All 19 events occurred within 4 months after alloSCT. The patient with mild skin GvHD remained event-free for at least 51 months after alloSCT. None of the 104 non-high risk patients received an early low-dose DLI. At 6 months after alloSCT, the cumulative incidence of clinically significant GvHD was 26% (95%-CI 15-37) and 5% (95%-CI 0-9) for the high risk patients scheduled for early low-dose DLI and the non-high risk patients, respectively (**Supplementary Figure 2**). All clinically significant GvHD in the high risk patients occurred after administration of the early low-dose DLI (but before standard DLI) of which 88% occurred in patients receiving DLI from an UD after an ATG-containing conditioning regimen.

**Table 1:** Baseline characteristics.

	Total cohort (n = 166)	Intention for early low-dose DLI (n = 62)	No intention for early low-dose DLI (n = 104)
Age at alloSCT (years)			
median (range)	63 (28–78)	64 (31-78)	63 (28-73)
Disease			
AML	133 (80%)	46 (74%)	87 (84%)
ALL	17 (10%)	10 (16%)	7 (7%)
MDS	16 (10%)	6 (10%)	10 (10%)
Nonmyeloablative conditioning			
Flu/Bu	150 (90%)*	46 (74%)	104 (100%)*
Flu/Bu/Ara-C/Amsa (FLAMSA)	16 (10%)	16 (26%)	0
Donor			
RD, 10/10 HLA matched	57 (34%)	20 (32%)	37 (36%)
UD, 10/10 HLA matched	101 (61%)	39 (63%)	62 (60%)
UD, 9/10 HLA matched	8 (5%)	3 (5%)	5 (5%)
Graft source			
G-CSF mobilized PBSC	165 (99%)	62 (100%)	103 (99%)
BM	1 (1%)	0	1 (1%)
CMV serostatus patient/donor			
+/+	79 (48%)	32 (52%)	47 (45%)
+/-	25 (15%)	8 (13%)	17 (16%)
-/+	11 (7%)	4 (6%)	7 (7%)
-/-	51 (31%)	18 (29%)	33 (32%)
Main reason for intention for early low-dose DLI			
FLAMSA regimen	-	16 (26%)	-
MRD+ at time of alloSCT	–	14 (23%)	–
AML/MDS: EVI1 overexpression	-	9 (15%)	-
AML: monosomal karyotype	–	8 (13%)	–
AML: ASXL mutation, only one remission induction course, or persisting underlying disease	-	4 (6%)	-
ALL: t(9;22)	–	4 (6%)	–
ALL: hypodiploidy, no CR1, or t(4;11)	-	4 (6%)	-
Therapy-related AML	–	2 (3%)	–
AML: progression before alloSCT	-	1 (2%)	-

*One patient had not received a second consolidation course before transplant and received 2 days cyclophosphamide 750 mg/m^2^ intravenously additionally to the conditioning regimen.

Intention for early low-dose DLI is based on the anticipated high risk of relapse after alloSCT. DLI, donor lymphocyte infusion; alloSCT, allogeneic stem cell transplantation; AML, acute myeloid leukemia; ALL, acute lymphoblastic leukemia; MDS, myelodysplastic syndrome; Flu, fludarabine; Bu, busulfan; Ara-C, cytarabine; Amsa, amsacrine; RD, related donor; UD, unrelated donor; G-CSF, granulocyte-colony stimulation factor; PBSC, peripheral blood stem cells; BM, bone marrow.

### 3.2 T-cell trajectories after alloSCT and DLI

#### 3.2.1 DLI-related increase of T-cell counts after 3 months after alloSCT observed in patients with an unrelated donor

**Figure 2:**
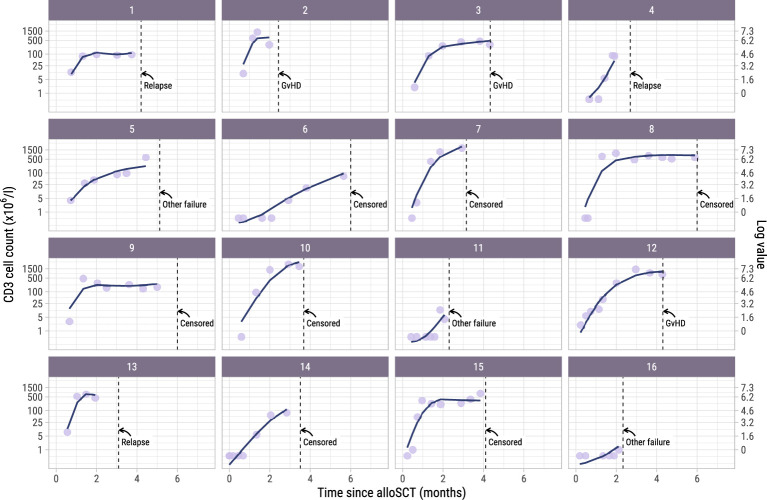
Observed versus estimated CD3 counts from alloSCT. Observed (dots) and estimated subject-specific trajectories (solid line) of a random subset of 16 patients in the dataset. The estimated trajectories are based on the longitudinal submodel of model I. Dotted lines show the time of terminating event or administrative censoring because of administration of a modified T-cell product or standard DLI. The secondary axis shows the cell counts on the log scale, which is the scale used for modelling. For example, a cell count of 1 on the primary axis corresponds to log(1) = 0 on the secondary axis.

**Figure 3:**
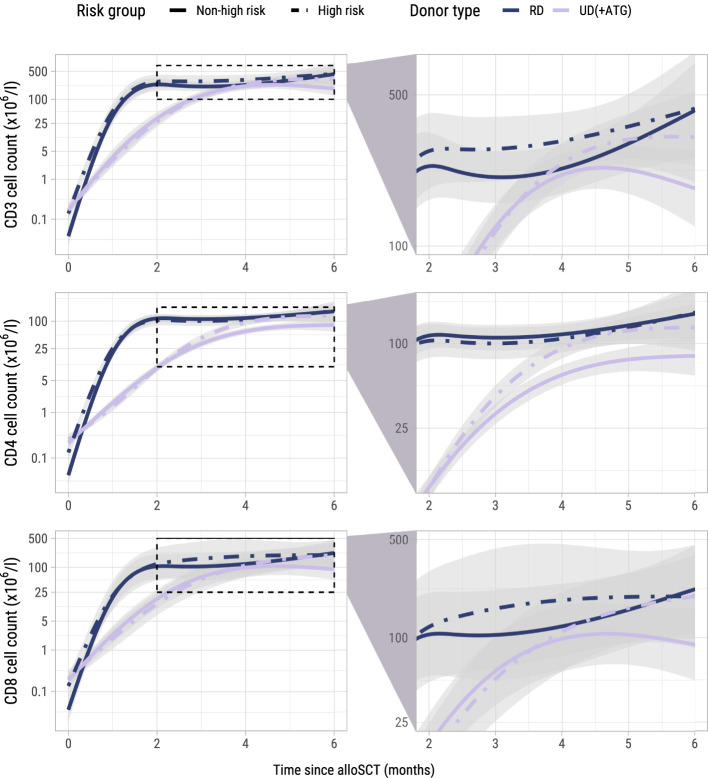
Model-based T-cell count trajectories after alloSCT. Predicted average trajectories of the total, CD4+ and CD8+ T-cell counts during the first 6 months after alloSCT, based on the longitudinal submodel of model I. For all predicted trajectories, the patient/donor CMV status was set to -/-. 95% confidence intervals are shown in grey. The right column zooms in on a specific part of the total trajectory.

To investigate whether administration of the early low-dose DLI increased the numbers of circulating T-cells during the first 6 months after alloSCT, we performed an ITT analysis using model I (see Methods) to compare the 62 high risk patients who were scheduled for early low-dose DLI with the 104 non-high risk patients who were not. All patients had at least 2 T-cell measurements with a median of 6 measurements per patient (interquartile range: 5-8). Although patients showed very different T-cell kinetics over time (**Supplementary Figure 3**), the model was flexible enough to capture the different shapes of patient-specific trajectories (**Figure 2**). Patients who were CMV seropositive or who had a CMV seropositive donor had significantly higher CD3 and CD8 counts during the first 6 months after TCD alloSCT compared to CMV seronegative patients with a CMV seronegative donor, corresponding to a significant increase on the log scale of 0.49 (95%-CI 0.31-0.67) and 0.45 (95%-CI 0.08-0.80) for CD3+ and CD8+ T-cells, respectively. For instance, the model-based CD3 count at 6 months for a non-high risk patient with a RD was 425x10^6^/l if CMV -/- compared to 694x10^6^/l for any other CMV serostatus combination. The model-based CD8 count at this time was 222x10^6^/l compared to 347x10^6^/l, respectively, suggesting expansion of CMV-specific T-cells. A same trend was observed for the CD4 counts (increase of 0.11 on the log scale, 95%-CI 0-0.23). As shown in **Figure 3**, patients with an UD had lower T-cell counts during the first 3 months after TCD alloSCT than patients with a RD, illustrating the enduring effect of the additional ATG that was given to all patients with an UD. We observed no significant difference in the cell count trajectories between the disease risk groups for patients with a RD. In contrast, in patients with an UD the CD4 trajectories started to diverge at 3 months after alloSCT, resulting in higher cell counts in the high risk patients intended to receive an early low-dose DLI at 3 months. The CD3 and CD8 counts showed similar trends. Taken together, these data show that a strategy of early low-dose DLI can lead to T-cell expansion.

#### 3.2.2 CD3, CD4 and CD8 counts increase after early low-dose DLI

**Figure 4:**
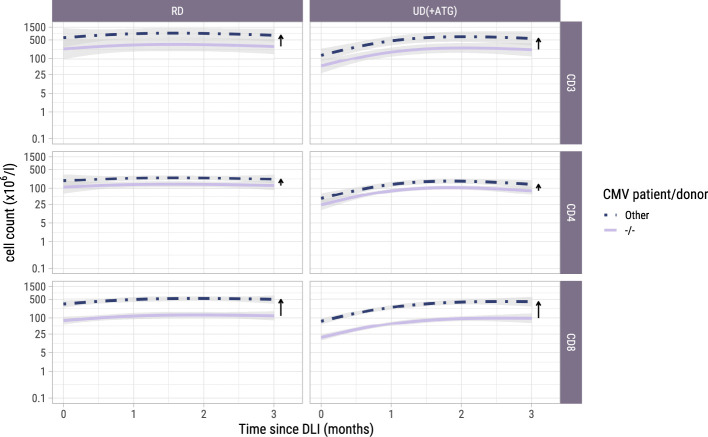
Model-based T-cell count trajectories after early low-dose DLI. Predicted average trajectories of the total, CD4+ and CD8+ T-cell counts during the first 3 months after early low-dose DLI. These are based on the longitudinal submodel of model II. 95% confidence intervals are shown in grey. The distance between the two lines in each panel (and further visualized by the adjacent arrows) corresponds to the CMV patient/donor effect on the trajectories. Namely, higher cell counts are predicted for patient/donor pairs where at least one is CMV seropositive, relative to a pair where both are CMV seronegative.

To investigate whether the T-cell counts increased after the early low-dose DLI as the ITT-analysis suggested, we used model II including only the 42 patients who actually received this DLI without any prior event and modelled the kinetics during the first 3 months after DLI. One of the 42 patients did not have any T-cell measurement during this period and was excluded. Baseline characteristics of the 41 included patients are described in **Supplementary Table 1**. These patients had at least one T-cell measurement during the 3-month period after early low-dose DLI with a median of 4 measurements (interquartile range: 2-5). Again, a flexible model was constructed to capture the different shapes of the T-cell kinetics of the included patients (**Supplementary Figure 4** and **Supplementary Figure 5**). The model-based trajectories of the total, CD4+ and CD8+ T-cell counts (**Figure 4**) showed increasing T-cell counts after DLI, with similar effects of the patient/donor CMV serostatus and donor type on the T-cell counts as in the earlier models.

### 3.3 Associations between T-cell kinetics and alloimmune responses after alloSCT and DLI

#### 3.3.1 Higher CD3 and CD4 counts are associated with a higher risk of GvHD

**Figure 5:**
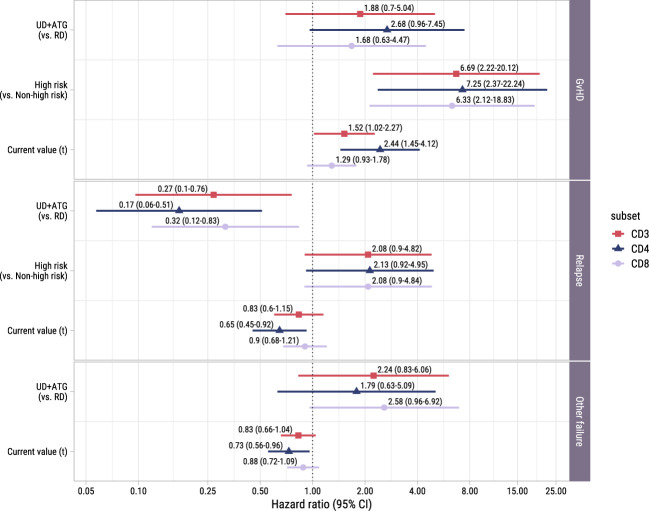
Forest plot for ITT analysis. Hazard ratios with associated 95% confidence intervals for donor type, disease risk and current value of the log of total, CD4+ or CD8+ T-cell counts on the events of interest. These are based on the time-to-event submodel of model I (see **Figure 1A**).

To study the association between the T-cell kinetics and the development of GvHD or relapse after TCD alloSCT and DLI, we added disease risk and donor type as time-fixed covariates alongside the time-dependent T-cell counts in the cause-specific submodels (with GvHD, relapse and other failure as competing events) of model I. As shown in **Figure 5**, donor type showed no significant association with the risk of GvHD, although in the CD4 model a trend for higher risk in patients with an UD despite the ATG in the conditioning regimen was observed (hazard ratio [HR] 2.7, 95%-CI 1.0-7.4). High risk patients, who were scheduled for early low-dose DLI, had a considerably higher risk of GvHD compared to non-high risk patients with HRs ranging between 6.3 (CD8 model, 95%-CI 2.1-18.8) and 7.3 (CD4 model, 95%-CI 2.4-22.2), indicating an alloimmune effect of the early low-dose DLI in this setting. The current values of the log CD4 and CD3 counts significantly increased the risk of GvHD (HR 2.4 (95%-CI 1.4-4.1) and HR 1.5 (95%-CI 1.0-2.3) for CD4+ T-cells and CD3+ T-cells, respectively), while CD8+ T-cells showed a similar trend (HR 1.3, 95%-CI 0.9-1.8). These HRs represent the relative increase in GvHD risk for an increase of one in the log counts, assuming same disease risk and donor type. These results indicate that the absolute total numbers of circulating CD4+ and CD3+ T-cells after alloSCT and DLI are informative for the development of GvHD.

We hypothesized that not only the current value but also the slope of the T-cell counts would be associated with the development of an alloimmune response. To investigate this, we extended the time-to-event submodel of model I by additionally including the current slope of the T-cell counts as a covariate for all endpoints. However, we observed no association between the slope of any of the T-cell subsets and the development of GvHD (p-values 0.59-0.87). We therefore retained the simpler version of model I with only the current value.

#### 3.3.2 Protective effect of CD4+ T-cells against relapse and other failure

To investigate whether higher T-cell counts were associated with a lower risk of relapse, we examined the risk factors for relapse in the time-to-event submodel of model I. Despite the ATG, patients with an UD had a significantly lower risk of relapse than patients with a RD (HRs ranging between 0.2 (95%-CI 0.1-0.5) and 0.3 (95%-CI 0.1-0.8), **Figure 5**). A trend was observed for higher relapse risk in the high risk patients (HR 2.1 in all models, 95%-CI for CD4+ T-cells: 0.9-5.0, respectively), suggesting that the addition of early low-dose DLI to the strategy did not completely compensate for the higher relapse risk. While CD3+ and CD8+ T-cells showed no significant association with relapse, higher CD4 counts decreased the risk of relapse significantly (HR 0.6, 95%-CI 0.5-0.9).

Of the 36 patients who experienced other failures, 6 died, 8 developed graft failure, 18 required systemic immunosuppression for a non-GvHD indication (of whom 9 received rituximab for EBV) and 4 received a virus-specific T-cell infusion for a severe viral infection. Only in the CD8 model a trend was observed for a higher risk of other failure in patients with an UD receiving an ATG-containing conditioning regimen (HR 2.6, 95%-CI 1.0-6.9). Higher CD4+ T-cell counts significantly lowered the hazard of the composite endpoint other failure (HR 0.7, 95%-CI 0.6-1.0).

#### 3.3.3 T-cell counts after early low-dose DLI retain their association with the development of GvHD

**Figure 6:**
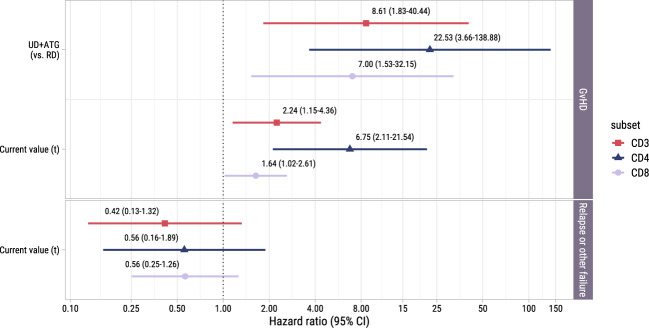
Forest plot for postDLI models. Hazard ratios with associated 95% confidence intervals for donor type and current value of the log of total, CD4+ or CD8+ T-cell counts on the events of interest. These are based on the time-to-event submodel of model II (see **Figure 1B**).

To investigate whether the T-cell kinetics were also associated with the development of alloimmune responses in the postDLI setting, we used the time-to-event submodel of model II starting from early low-dose DLI with GvHD and non-GvHD events as competing events. We observed no significant association between the current values and the very heterogenous composite endpoint of relapse and other failure (**Figure 6**). However, patients with an UD had a considerably higher risk of GvHD with HRs ranging between 7.0 (CD8+ T-cells, 95%-CI 1.5-32.1) and 22.5 (CD4+ T-cells, 95%-CI 3.7-138.9) compared to patients with a RD. For all T-cell subsets, higher current values increased the risk of GvHD with HRs ranging between 1.6 (CD8+ T-cells, 95%-CI 1.0-2.6) and 6.7 (CD4+ T-cells, 95%-CI 2.1-21.5). These data show that in the subset of patients receiving early low-dose DLI, total CD3+, CD4+ and CD8+ T-cell counts after DLI are associated with the development of GvHD.

### 3.4 NK cell kinetics and associations with alloimmune responses after alloSCT

**Figure 7:**
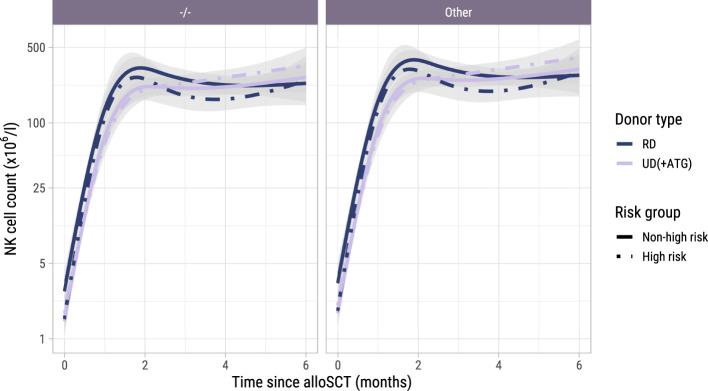
Model-based NK cell count trajectories after alloSCT. Predicted average trajectories of the NK cell counts during the first 6 months after alloSCT, based on the longitudinal submodel of model I. The left panel shows the predicted trajectories for CMV seronegative patients with a CMV seronegative donor, the right panel the predicted trajectories for patients with any other patient/donor CMV serostatus combination. 95% confidence intervals are shown in grey.

To investigate the NK cell kinetics and their association with GvHD and relapse, we returned to model I starting at alloSCT. As shown in **Supplementary Figure 6**, the NK cell counts recovered rapidly, reaching the normal levels of 40-390x10^6^ NK cells/l for almost all patients within 2 months, before the time of administration of the early low-dose DLI. As shown in **Figure 7**, CMV seropositive patients or patients with a CMV seropositive donor had significantly higher NK counts than CMV -/- patients, as was seen for the T-cell subsets. In contrast to T-cell kinetics, patients with an UD and ATG did not have a slower recovery of NK counts compared to patients with a RD and no ATG. Furthermore, there was no association between the risk group and NK counts, indicating that there was no impact of DLI on the NK cell kinetics. Higher current NK counts were associated with a higher risk of GvHD (HR 1.95 per unit log count increase, 95%-CI 1.10-3.47) and a lower risk of relapse (HR 0.62, 95%-CI 0.41-0.93) but had no significant association with the risk of other failure. We hypothesized that the observed association between the NK count and GvHD may not be due to a direct effect of the NK cells, but instead reflected the high correlation between the NK and CD4 count trajectories, the latter being expected to be the main driver of GvHD. We therefore ran a cause-specific Cox model for GvHD, which included disease risk and donor type as time-fixed covariates, and *both* CD4 and NK counts as time-dependent covariates. In this model, CD4 counts were significantly associated with the development of GvHD (HR 2.08, 95%-CI 1.16-3.74) while the HR for the NK cell counts was 1.07 (p-value 0.83), supporting that the CD4+ T-cells were the important drivers for the development of GvHD.

## 4 Discussion

In this study we investigated the interplay between immune cell kinetics and alloimmune responses after both TCD alloSCT and subsequent DLI using joint modelling. In the ITT analysis we observed significantly more GvHD in the high risk patients intended to receive an early low-dose DLI and an increase in T-cell counts starting at 3 months after alloSCT in high risk patients with an UD receiving an ATG-containing conditioning regimen. The ITT allocation was solely based on the disease characteristics of the patients. Since all patients were in complete remission at time of alloSCT, the TCD strategy was similar between the disease risk groups, and all GvHD in the high risk group only occurred after DLI, the only plausible explanation for both the higher risk of GvHD and the associated T-cell expansion is the administration of the early low-dose DLI. We also observed significant associations between the CD4 counts and alloimmune responses after TCD alloSCT and DLI: an increase in CD4+ T-cells was associated with a higher risk of GvHD and at the same time a lower risk of relapse suggesting establishment of a GvL effect. Interestingly, we only observed DLI-induced T-cell expansion in patients transplanted using an UD. This likely reflects an alloimmune response as GvHD was mainly seen in patients with an UD after receiving a DLI, and the T-cell counts after DLI were associated with the development of GvHD. The alloreactive T-cell expansion may have been more easily detectable in patients with an UD compared to RD because of the deeper lymphopenia at time of DLI due to the long-lasting immunosuppressive effect of ATG that patients with an UD received (13). In addition, the high prevalence of HLA-DP mismatches, targeted by CD4+ T-cells, in patients with an UD (32–34) could contribute to the strong association between CD4+ T-cells and the development of GvHD. In contrast to T-cells, NK cells recovered early after alloSCT and were not significantly influenced by donor type and TCD, consistent with previous studies (13, 35, 36), nor by DLI. As previously reported (3, 37), higher NK counts were associated with a lower risk of relapse. The joint model also suggested that higher NK counts were associated with a higher risk of GvHD. However, in an exploratory cause-specific Cox model, this association between NK cells and GvHD disappeared after adjusting for the CD4 counts, indicating that the CD4+ T-cells were the important drivers for GvHD.

Our results suggest a DLI-induced T-cell expansion measurable in total numbers of the major T-cell subsets where others did not observe a significant effect of DLI on the T-cell kinetics (18–21). This may be due to several factors. Our comparatively larger cohort size (other studies usually included less than 25 patients) allowed for detection of more subtle differences. Furthermore, the strategy of administering early prophylactic DLI to a subset of patients based on their relapse risk provided an intervention and control group who were treated according to the same transplantation strategy. Lastly, conclusions drawn can be influenced by the choice of the statistical method. For example, matched pair analysis as used by Guillaume et al. (19) and Schultze-Florey et al. (21) only allowed them to compare the cells counts between two timepoints. The repeated measures analysis used by Nikiforow et al. (20) and the mixed model used by Bullucini et al. (18) allowed to compare the trajectories over time but could not account for informative dropout. Because we used joint modelling, we could flexibly model the T-cell trajectories over a longer period of time and properly account for informative dropout and random variation. To our knowledge, thus far only a single study used joint modelling to study T-cell kinetics after alloSCT (38). We now have used this technique to investigate the immunological effects of DLI.

There are several limitations to our study. The total CD3, CD4 and CD8 counts are crude measures for potentially alloreactive T-cells, as only donor-derived T-cells can induce GvHD and GvL and the counts are not informative about the subpopulations, activation status or kinetics of specific T-cell clones. Thus, if we had measured the chimerism status and clonality, we might have expected to find stronger associations between the T-cell kinetics and the clinical events. Moreover, our ITT approach attenuated the observed effects of DLI on the T-cell kinetics and clinical endpoints as not all high risk patients received the early low-dose DLI and most patients who did receive this DLI did not receive it at exactly the same time after transplant. Therefore, we constructed model II starting from early low-dose DLI to see whether similar associations were observed. Joint modelling requires substantial numbers of both clinical events and longitudinal measurements to estimate associations with sufficient accuracy. Despite our comparatively larger sample size, the modest numbers of clinical events limited both the accurate estimation of association parameters (between T-cell counts and the endpoints), as well as the inclusion of additional risk factors for each endpoint. This was especially noticeable in our models focusing on the subset of the patients actually receiving an early low dose DLI. Due to the limited number of events, we used suboptimal composite endpoints such as ‘other failure’ and ‘relapse and other failure’, which hampered estimation of the association between the T-cell kinetics and these endpoints.

Further studies are necessary to assess the clinical implications of the findings from the present work. Aside from validation of our findings, larger studies must be performed to investigate the predictive utility of the T-cell and NK cell counts. While these counts are crude measures, they are often measured standardly and therefore attractive biomarkers for predicting alloimmune responses in patients receiving alloSCT and/or DLI. Further investigation of the immune cell kinetics in other alloSCT settings is needed to see whether similar associations between the T-cell and NK cell kinetics and alloimmune responses can be observed when using joint modelling. For instance, the recent machine learning analysis by McCurdy et al. also suggested important roles of CD4+ T-cells in the development of acute GvHD and of NK cells in the development of relapse after alloSCT with posttransplant cyclophosphamide (37). For DLI, we would suggest to perform a prospective study where the T-cell counts are measured at time of DLI and every week after DLI during the first 6 weeks. Most GvHD develops within this period and by measuring more often, dynamic prediction tools (i.e. updated personalized probabilities of GvHD given measurement history) could be developed (39). In order to develop such tools however, one would ideally need to model the T-cell subsets and NK cells *jointly* as part of a multivariate joint model, which will account for the correlation between each subset, but may be complicated to fit and will require larger sample sizes. In our study, we were not able to present such a multivariate joint model because of both sample size and software limitations. Nevertheless, results from the exploratory time-dependent cause-specific Cox model for GvHD with both the CD4 and NK counts hint at the importance of modelling immune subsets jointly.

Generally speaking, further characterization of the circulating T-cell subsets, differentiation and metabolic fitness could provide valuable additional insight in future studies on T-cell kinetics (40, 41).

In summary, joint modelling allowed us to capture the associations between DLI, T-cell and NK cell counts, GvHD and relapse in a very complex clinical setting, even with modest numbers of patients and events. NK cells recover early after alloSCT and may have a protective effect against relapse. We demonstrate that DLI can induce detectable T-cell expansion and observe that the CD4+ T-cells show the strongest association with the development of alloimmune responses. Higher CD4 counts increase the risk of GvHD and decrease the risk of relapse.

## Data availability statement

The raw data supporting the conclusions of this article will be made available by the authors, without undue reservation.

## Ethics statement

The studies involving human participants were reviewed and approved by Medical Ethical Committee of Leiden University Medical Center. The patients/participants provided their written informed consent to participate in this study.

## Author contributions

EK, EB, LW, CH, HP and JF designed the study. EK and EB collected and prepared the data, and performed the data analyses under supervision of LW, CH, HP and JF. All authors helped interpreting the data. EK and EB wrote the manuscript with contributions from LW, CH, HP and JF. PvdB, PvB, EM, JT, TS, DL, HV, JF and CH provided the patients care. All authors critically reviewed and approved the manuscript.
