# Correction: Joint models quantify associations between immune cell kinetics and allo-immunological events after allogeneic stem cell transplantation and subsequent donor lymphocyte infusion

**DOI:** 10.3389/fimmu.2025.1637094

**Published:** 2025-06-13

**Authors:** Eva A. S. Koster, Edouard F. Bonneville, Peter A. von dem Borne, Peter van Balen, Erik W. A. Marijt, Jennifer M. L. Tjon, Tjeerd J. F. Snijders, Daniëlle van Lammeren, Hendrik Veelken, Hein Putter, J. H. Frederik Falkenburg, Constantijn J. M. Halkes, Liesbeth C. de Wreede

**Affiliations:** ^1^ Department of Hematology, Leiden University Medical Center, Leiden, Netherlands; ^2^ Department of Biomedical Data Sciences, Leiden University Medical Center, Leiden, Netherlands; ^3^ Department of Hematology, Medisch Spectrum Twente, Enschede, Netherlands; ^4^ Department of Hematology, HagaZiekenhuis, The Hague, Netherlands

**Keywords:** T-cell kinetics, joint modelling, allogeneic stem cell transplantation, donor lymphocyte infusion, graft-versus-host-disease, T-cell depletion, acute myeloid leukemia, acute lymphoblastic leukemia

In the published article, an author surname was erroneously mentioned as “Borne” instead of “von dem Borne” in the citation and copyright sections. The citation and copyright have been corrected as shown below:

“Citation:

Koster EAS, Bonneville EF, von dem Borne PA, van Balen P, Marijt EWA, Tjon JML, Snijders TJF, van Lammeren D, Veelken H, Putter H, Falkenburg JHF, Halkes CJM and de Wreede LC (2023) Joint models quantify associations between immune cell kinetics and allo-immunological events after allogeneic stem cell transplantation and subsequent donor lymphocyte infusion. Front. Immunol. 14:1208814. doi: 10.3389/fimmu.2023.1208814

Copyright:

**©** 2023 Koster, Bonneville, von dem Borne, van Balen, Marijt, Tjon, Snijders, van Lammeren, Veelken, Putter, Falkenburg, Halkes and de Wreede”

The original article has been updated.

