# Supplementary material for: Joint models quantify associations between immune cell kinetics and allo-immunological events after allogeneic stem cell transplantation and subsequent donor lymphocyte infusion
Source: Front Immunol. 2023 Aug 1;14:1208814. doi: 10.3389/fimmu.2023.1208814 (PMC10427852; doi:10.3389/fimmu.2023.1208814)
Supplement: Supplementary file 1 [file DataSheet_1.pdf]

Supplementary Tables and Figures

Joint models quantify associations between immune cell kinetics and allo-immunological events after allogeneic stem cell transplantation and subsequent donor lymphocyte infusion

Eva A.S. Koster*, Edouard F. Bonneville, Peter A. von dem Borne, Peter van Balen, Erik W.A. Marijt, Jennifer M.L. Tjon, Tjeerd J.F. Snijders, Daniëlle van Lammeren, Hendrik Veelken, Hein Putter, J.H. Frederik Falkenburg, Constantijn J.M. Halkes, Liesbeth C. de Wreede

*** Correspondence:** Eva A.S. Koster: e.a.s.koster@lumc.nl

1 Supplementary Tables

	**Early low-dose DLI (n=41)**
**Age at alloSCT (years)**	
median (range)	65 (31-74)
**Disease**	
AML	29 (71%)
ALL	8 (20%)
MDS	4 (10%)
**Nonmyeloablative conditioning**	
Flu/Bu	30 (73%)
Flu/Bu/Ara-C/Amsa (FLAMSA)	11 (27%)
**Donor**	
RD, 10/10 HLA matched	12 (29%)
UD, 10/10 HLA matched	27 (66%)
UD, 9/10 HLA matched	2 (5%)
**Graft source**	
G-CSF mobilized PBSC	42 (100%)
**CMV serostatus patient/donor**	
+/+	17 (41%)
+/-	5 (12%)
-/+	3 (7%)
-/-	16 (39%)
**Reason for early low-dose DLI**	
Conditioning using the FLAMSA regimen	11 (27%)
MRD+ at time of alloSCT	10 (24%)
ALL: t(9;22)	3 (7%)
ALL: t(4;11), hypodiploidy, or not in CR1	3 (7%)
AML: monosomal karyotype	5 (12%)
AML/MDS: EV1 overexpression	6 (15%)
AML: ASXL mutation, only 1 intensive remission induction course, or persisting CMML	3 (7%)

Supplementary Table 1. Baseline characteristics of the 41 evaluable patients with early low-dose DLI. DLI, donor lymphocyte infusion; alloSCT, allogeneic stem cell transplantation; AML, acute myeloid leukemia; ALL, acute lymphoblastic leukemia; MDS, myelodysplastic syndrome; Flu, fludarabine; Bu, busulfan; Ara-C, cytarabine; Amsa, amsacrine; RD, related donor; UD, unrelated donor; G-CSF, granulocyte-colony stimulation factor; PBSC, peripheral blood stem cells; BM, bone marrow; MRD, minimal residual disease; CR1, first complete morphological remission; CMML, chronic myelomonocytic leukemia

2 Supplementary Figures

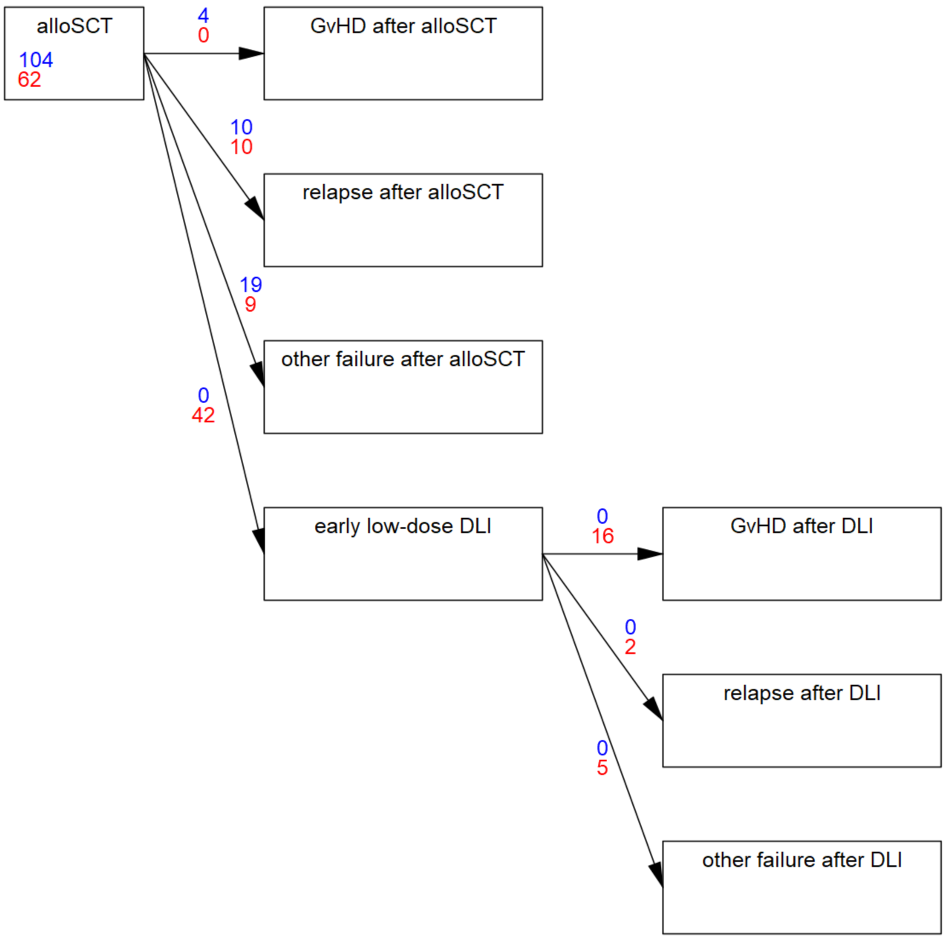

Supplementary Figure 1. Flow diagram of events during the first 6 months after alloSCT. Flow diagram of the events of interest after alloSCT and early low-dose DLI. The numbers in the top left box show the total numbers of included high risk patients scheduled for early low-dose DLI (red) and non-high risk patients (blue). The numbers next to the arrows show the numbers of the patients who had the respective event during the first 6 months after alloSCT without any prior administration of a modified T-cell product or standard DLI (blue: non-high risk, red: high risk). For instance, all high-risk patients received an early low-dose DLI or developed clinically significant GvHD, relapse or other failure before this DLI could be administered, except one patient who only had mild GvHD and did not need any systemic immunosuppression: therefore, the red numbers along the leftmost set of arrows add up to 61 while 62 started in the left box.

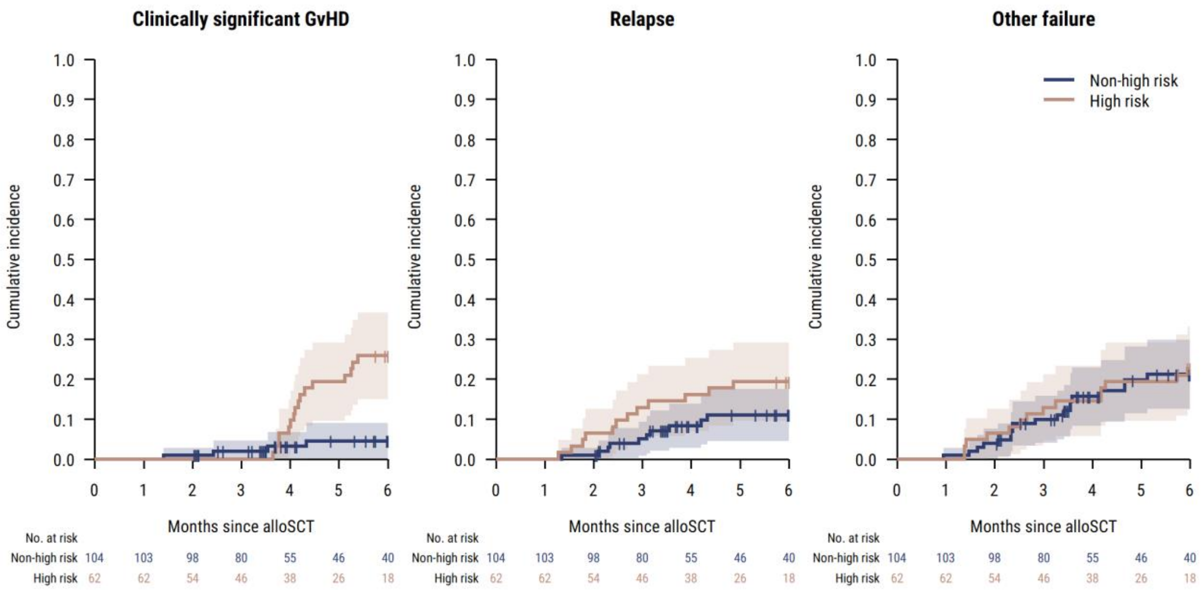

Supplementary Figure 2. Cumulative incidence of GvHD, relapse and other failure per disease risk group. Cumulative incidence of the competing events GvHD, relapse and other failure with associated 95% confidence intervals stratified by disease risk. Patients with a high anticipated risk of relapse were scheduled to receive an early low-dose DLI at 3 months after alloSCT. Contrary to **Supplementary Figure 1**, early low-dose DLI was not treated as an event in this figure. Patients who received a modified T-cell product or standard DLI were censored at 7 days after this DLI, indicated by |.

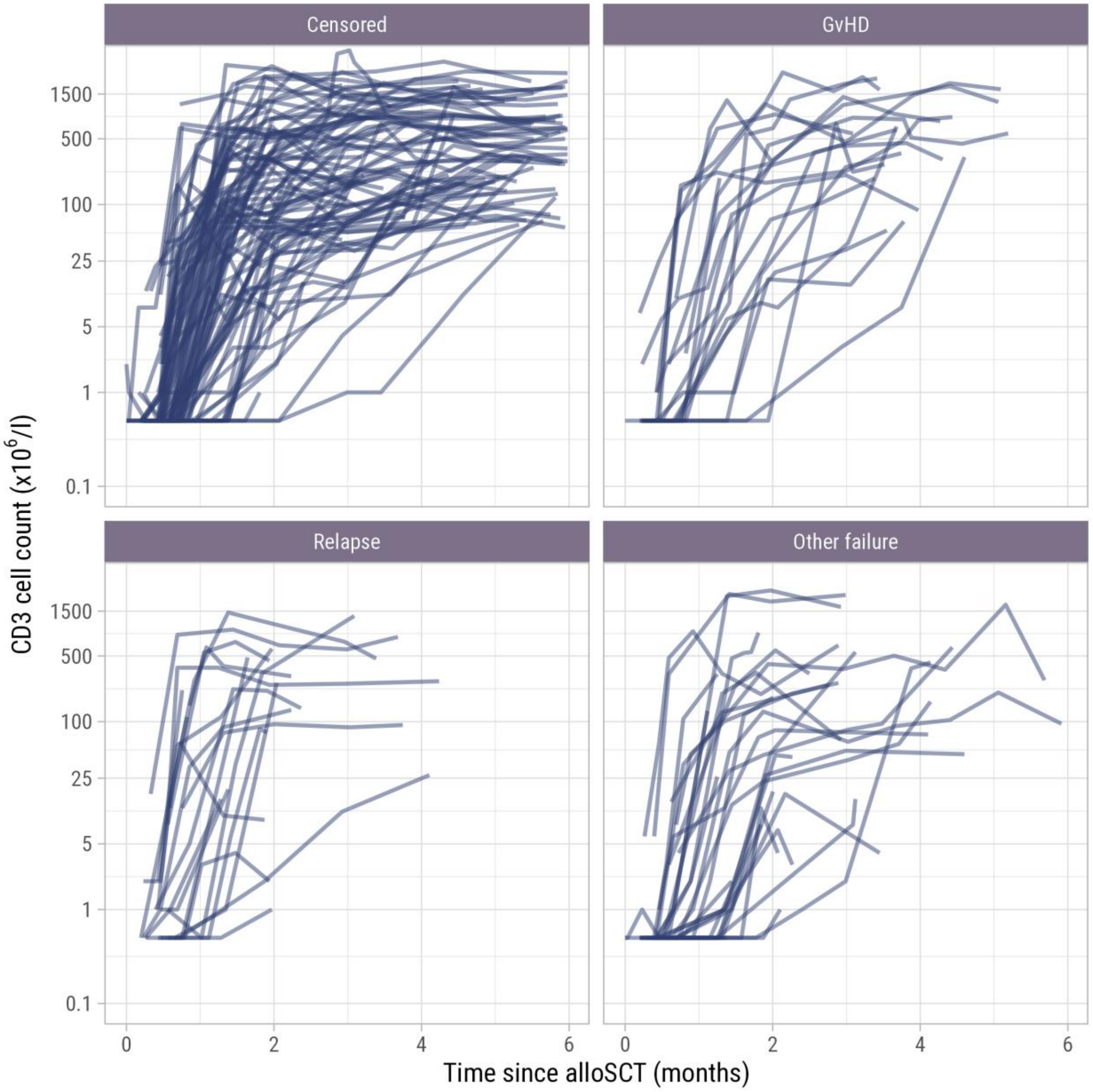

Supplementary Figure 3. Trajectories of total T-cell counts from alloSCT per terminating event. All observed trajectories for the CD3 counts during the first 6 months after alloSCT per terminating event. Patients were censored at 6 months after alloSCT, or 7 days after administration of a standard DLI or modified T-cell product, whichever occurred first. There was no loss to follow-up.

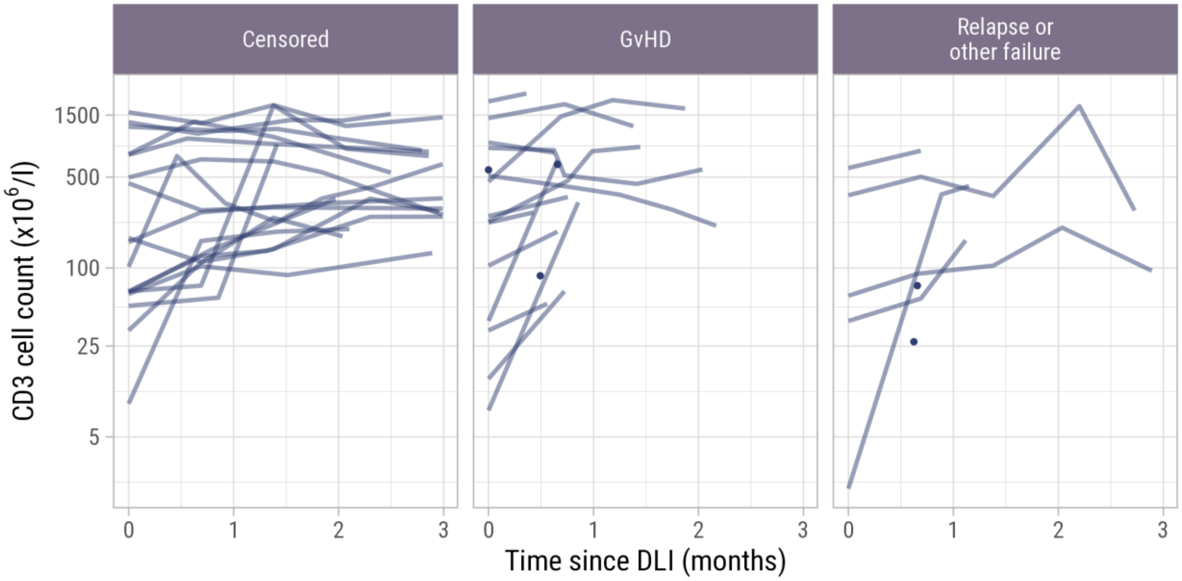

Supplementary Figure 4. Trajectories total T-cell counts after early low-dose DLI per terminating event. All observed trajectories for the CD3 counts during the first 3 months after early low-dose DLI per terminating event. The single points correspond to patients with only a single measurement between their DLI and terminating event. Patients were censored at 6 months after alloSCT, or 7 days after administration of a standard DLI or modified T-cell product, whichever occurred first. There was no loss to follow-up.

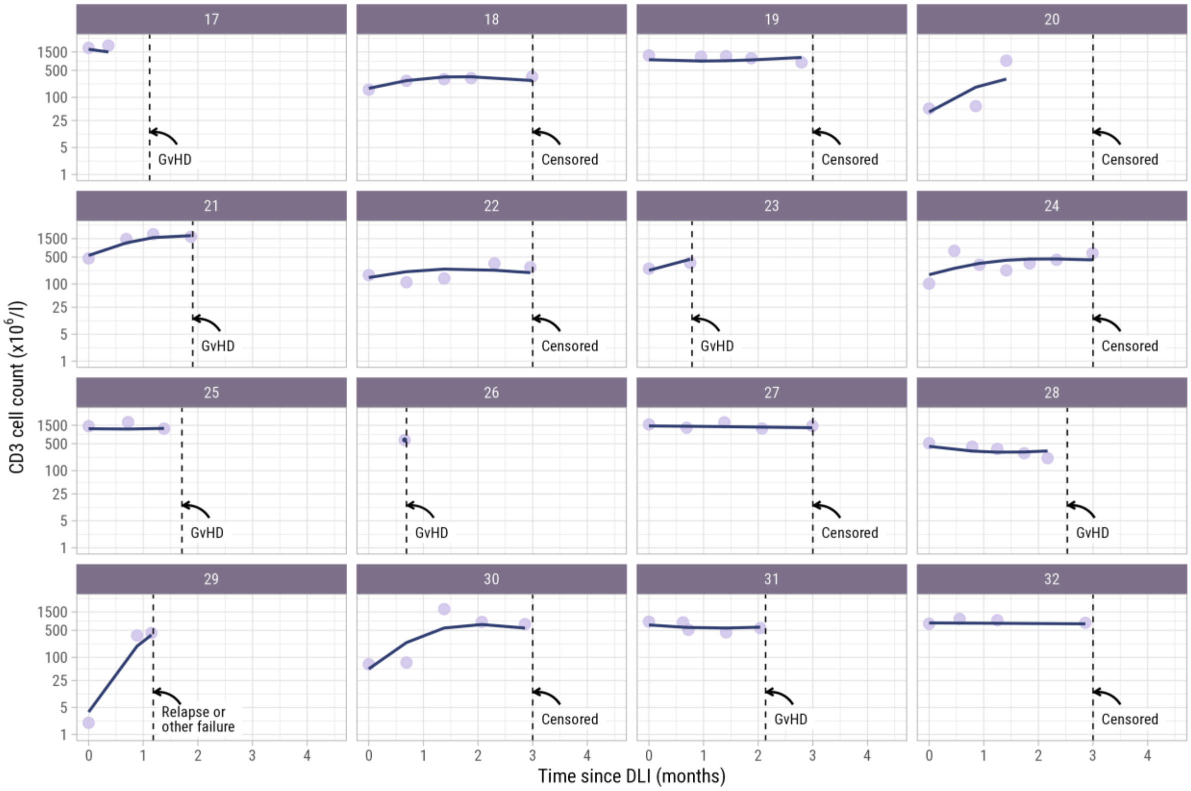

Supplementary Figure 5. Observed versus estimated CD3 counts after early low-dose DLI. Observed (dots) and estimated subject-specific trajectories (solid lines) of a random subset of 16 patients in the dataset. The estimated trajectories are based on the longitudinal submodel of model II. Dotted lines show the time of terminating event or administrative censoring because of administration of a modified T-cell product or standard DLI at 6 months after alloSCT.

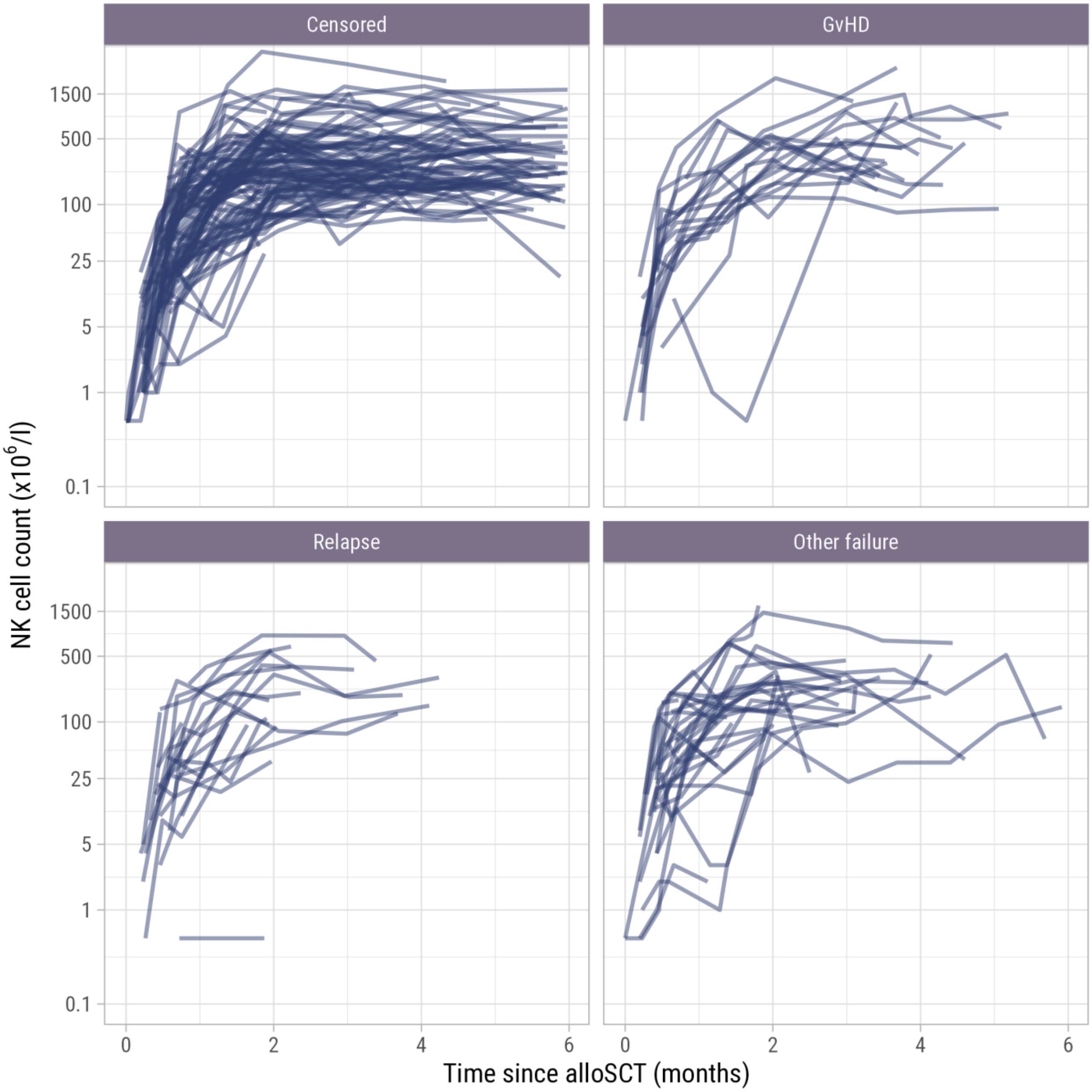

Supplementary Figure 6. Trajectories of NK cell counts from alloSCT per terminating event. All observed trajectories for the NK counts during the first 6 months after alloSCT per terminating event. Patients were censored at 6 months after alloSCT, or 7 days after administration of a standard DLI or modified T-cell product, whichever occurred first. There was no loss to follow-up.
