# Supplementary material for: Joint models quantify associations between immune cell kinetics and allo-immunological events after allogeneic stem cell transplantation and subsequent donor lymphocyte infusion
Source: Front Immunol. 2023 Aug 1;14:1208814. doi: 10.3389/fimmu.2023.1208814 (PMC10427852; doi:10.3389/fimmu.2023.1208814)

Statistical Supplement

Joint models quantify associations between immune cell kinetics and allo-immunological events after allogeneic stem cell transplantation and subsequent donor lymphocyte infusion

Eva A.S. Koster, Edouard F. Bonneville, Peter A. von dem Borne, Peter van Balen, Erik W.A. Marijt, Jennifer M.L. Tjon, Tjeerd J.F. Snijders, Daniëlle van Lammeren, Hendrik Veelken, Hein Putter, J.H. Frederik Falkenburg, Constantijn J.M. Halkes, Liesbeth C. de Wreede

---

The present supplementary material is a 'Statistical Supplement' to the main article, providing mathematical summaries of the models used.

1 JOINT MODEL I

Joint models only consider measurements taken prior to the occurrence of the clinical events of interest. Occasionally, the measurement time and event time coincide: for example, T-cell counts may be recorded on the same day as the start of therapeutic systemic immunosuppression for Graft-versus-Host-Disease (GvHD). In order to retain the information of the measurements taken at event times, we set the time of these measurements to one day earlier, which assumes that the measurement at the event time was representative of the T-cell counts the day before the event. However, we excluded measurements at time of relapse, since the presence of blasts in the peripheral blood could lead to incorrect counts of the normal T-cells. We also excluded measurements at time of autologous recovery, as donor-derived T-cells were no longer present, and therefore also no potentially alloreactive T-cells capable of inducing GvHD or Graft-versus-leukemia (GvL) effect.

1.1 Model formulation

The longitudinal submodel assumes that the true underlying (log) immune cell counts (either CD3, CD4, CD8, or NK) for the ith patient are given by

$$\begin{aligned} m_i(t) = \beta_0 &+ \sum_{q=1}^{3}(\beta_q + b_{iq})B_q(t) + \sum_{q=1}^{3}\beta_{q+3}\{B_q(t) \times \text{Risk}_i\} + \sum_{q=1}^{3}\beta_{q+6}\{B_q(t) \times \text{Donor}_i\} \\ &+ \sum_{q=1}^{3}\beta_{q+9}\{B_q(t) \times \text{Risk}_i \times \text{Donor}_i\} + \beta_{13}\text{CMV}_i + \beta_{14}\text{Risk}_i + \beta_{15}\text{Donor}_i \\ &+ \beta_{16}\{\text{Risk}_i \times \text{Donor}_i\}, \end{aligned}$$

with random effects vector $b_i \sim \mathcal{N}(0, D)$. The observations for the ith patient at timepoints t_{ij} $(j = 1, \ldots, n_i)$ are given by

$$y_{ij} = m_i(t_{ij}) + \epsilon_{ij},$$

where $\epsilon_{ij} \sim \mathcal{N}(0, \sigma^2)$ are independent random error terms.

Risk_i, Donor_i and CMV_i respectively represent the dummy variables for baseline disease risk (the intention-to-treat variable, high-risk compared to non-high risk), donor type (unrelated compared to related donor) and patient/donor Cytomegalovirus (CMV) serostatus at baseline (any one of patient or donor positive, compared to patient and donor both negative).

Time since allogeneic stem cell transplantation (alloSCT) was modelled flexibly assuming restricted (natural) cubic splines with two internal knots placed at the 33.3% and 66.7% percentiles of the measurement times. This is represented above by $B_q(t)$, corresponding to the qth basis function of the spline. The fixed effects part of the model posits a three-way interaction between time, donor type and baseline disease risk, as well as a main effect of patient/donor CMV status. The three-way interaction was constructed to a) capture the slower expected average trajectory of patients with an unrelated donor, due to the use of anti-thymocyte globulin (ATG) in this group; and b) to test for a difference in average trajectories between baseline disease risk groups.

In terms of random effects, this models assumes random slopes b_{iq} (one for each basis function), and a fixed intercept. This fixed intercept was justified given that this cohort underwent T-cell depleted (TCD) alloSCT, and all patients were therefore expected to start follow-up with immune cell counts close to zero. The random slopes were assumed to be normally distributed with mean zero, with unstructured covariance matrix D.

The time-to-event submodel was composed of multiple cause-specific proportional hazards models as

$$\begin{aligned} h_{1i}(t) &= h_{10}(t) \exp\Big\{\gamma_{11}\text{Donor}_i + \gamma_{12}\text{Risk}_i + \alpha_1 m_i(t)\Big\}, \\ h_{2i}(t) &= h_{20}(t) \exp\Big\{\gamma_{21}\text{Donor}_i + \gamma_{22}\text{Risk}_i + \alpha_2 m_i(t)\Big\}, \\ h_{3i}(t) &= h_{30}(t) \exp\Big\{\gamma_{31}\text{Donor}_i + \alpha_3 m_i(t)\Big\}, \end{aligned}$$

where the $h_{ki}(t)$ for $k \in \{1, 2, 3\}$ respectively represent the cause-specific hazards of GvHD, relapse, and other failures. The cause-specific baseline hazards $h_{k0}(t)$ were approximated on the log scale using cubic B-splines with three internal knots. The above corresponds to the 'current value' parametrization of the joint model, where the $\exp(\alpha_k)$ would represent the hazard ratio (for cause k) when comparing two patients (with same covariates) whose 'true' (model-based) underlying log immune cell values at a particular timepoint $m_i(t)$ differ by one. The γ_{kp} coefficients are interpreted analogously to main effects in standard cause-specific Cox proportional hazards models.

In addition to the current value parametrization, we also ran the models assuming a time-dependent slopes association structure as $\alpha_{k1} m_i(t) + \alpha_{k2}\{dmi(t)/dt\}$.

1.2 Goodness of fit

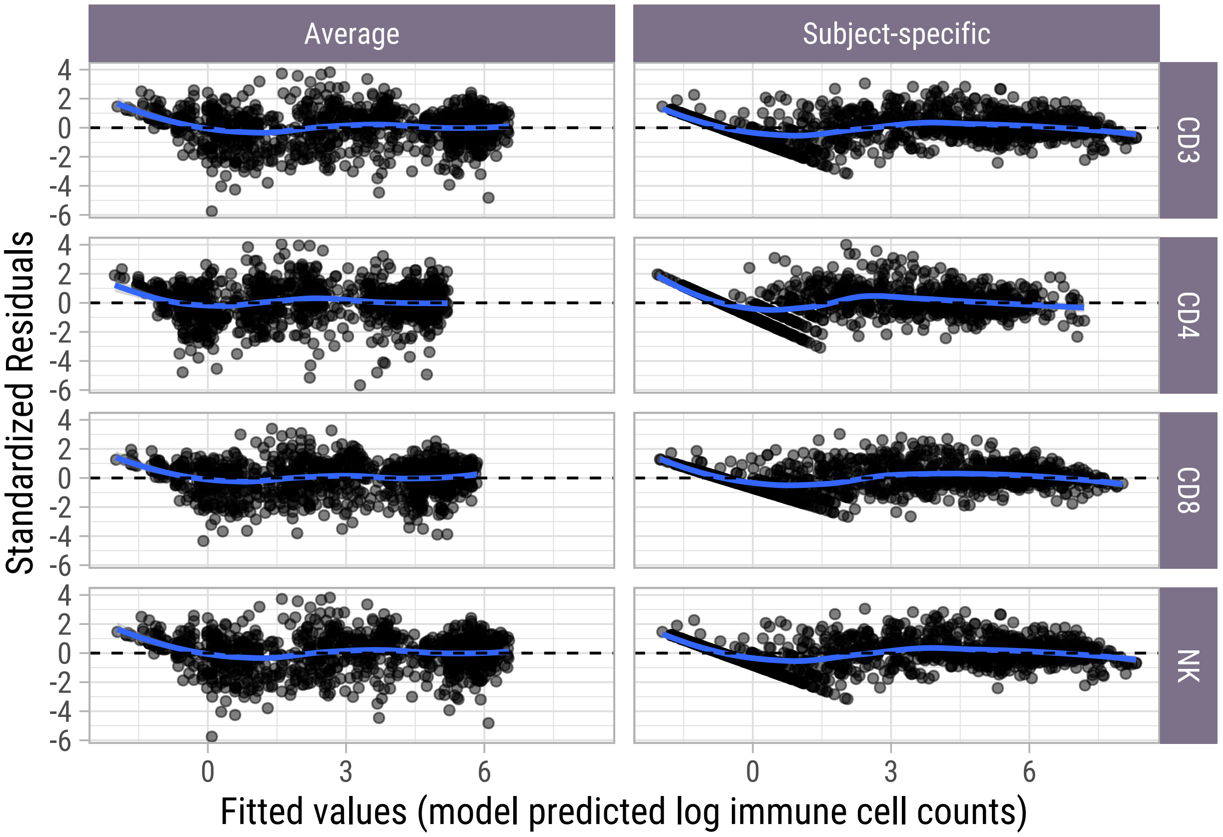

Above we present standardized residuals plots, which summarize how well the model fits the data overall (i.e. across all observations) - both for the average and subject-specific trajectories. The fitted (i.e. log immune cell counts predicted by the model) values are plotted against the standardized distance between the observed measurement and the predicted value. The blue line is a smoothed average of the standardized residuals as a function of the fitted values, and should ideally be horizontal at 0.

2 JOINT MODEL II

2.1 Model formulation

For model II, the time scale was no longer from alloSCT, but instead from time of early low-dose donor lymphocyte infusion (DLI). Therefore, this model was only run among the subset that *did* in fact receive an early low-dose DLI before the occurrence of other competing events. Furthermore, some patients did not have a T-cell measurement on the day of DLI but only a few days prior. For these patients, we used the measurement closest to DLI taken within the last week before DLI as the measurement at time of DLI (time 0).

The longitudinal submodel was again a linear mixed-effects model, where the true underlying log T-cell counts are given by

$$m_i(t) = (\beta_0 + b_{i0}) + \sum_{q=1}^{2} (\beta_q + b_{iq}) B_q(t) + \sum_{q=1}^{2} \beta_{q+2} \{B_q(t) \times \text{Donor}_i\} + \beta_5 \text{CMV}_i,$$

with random effects vector $b_i \sim \mathcal{N}(0, D)$. Observations for i^{th} patient are again given by

$$y_{ij} = m_i(t_{ij}) + \epsilon_{ij},$$

where $\epsilon_{ij} \sim \mathcal{N}(0, \sigma^2)$ are independent random error terms.

Time was again modelled with restricted cubic splines, but in contrast to model I, we used a single internal knot. The focus on a shorter timespan resulted in a reduced sample size, and fewer measurements per person. For consistency with model I, this average trajectory was allowed to differ across donor types (two-way interaction). In this model, disease risk at baseline was redundant as we ran the model among those having actually received an early low-dose DLI. A fixed effect for patient/donor CMV serostatus was also added to the model. This model comprised both random intercepts b_{i0} and random slopes b_{iq}, assumed to follow normal distributions with mean zero and unstructured covariance matrix.

Due to a limited number of events, relapse and other failures were merged into a composite endpoint. The time-to-event submodel was therefore specified as

$$\begin{aligned} h_{1i}(t) &= h_{10}(t) \exp\left\{\gamma_{11}\text{Donor}_i + \alpha_1 m_i(t)\right\}, \\ h_{2i}(t) &= h_{20}(t) \exp\left\{\alpha_2 m_i(t)\right\}, \end{aligned}$$

where the $h_{ki}(t)$ for $k \in \{1, 2\}$ respectively represent the cause-specific hazards of GvHD and the composite of relapse and other failures for subject i. The cause-specific baseline hazards $h_{k0}(t)$ were approximated on the log scale using cubic B-splines with two internal knots. In this joint model, only the current value parametrization was explored.

2.2 Goodness of fit

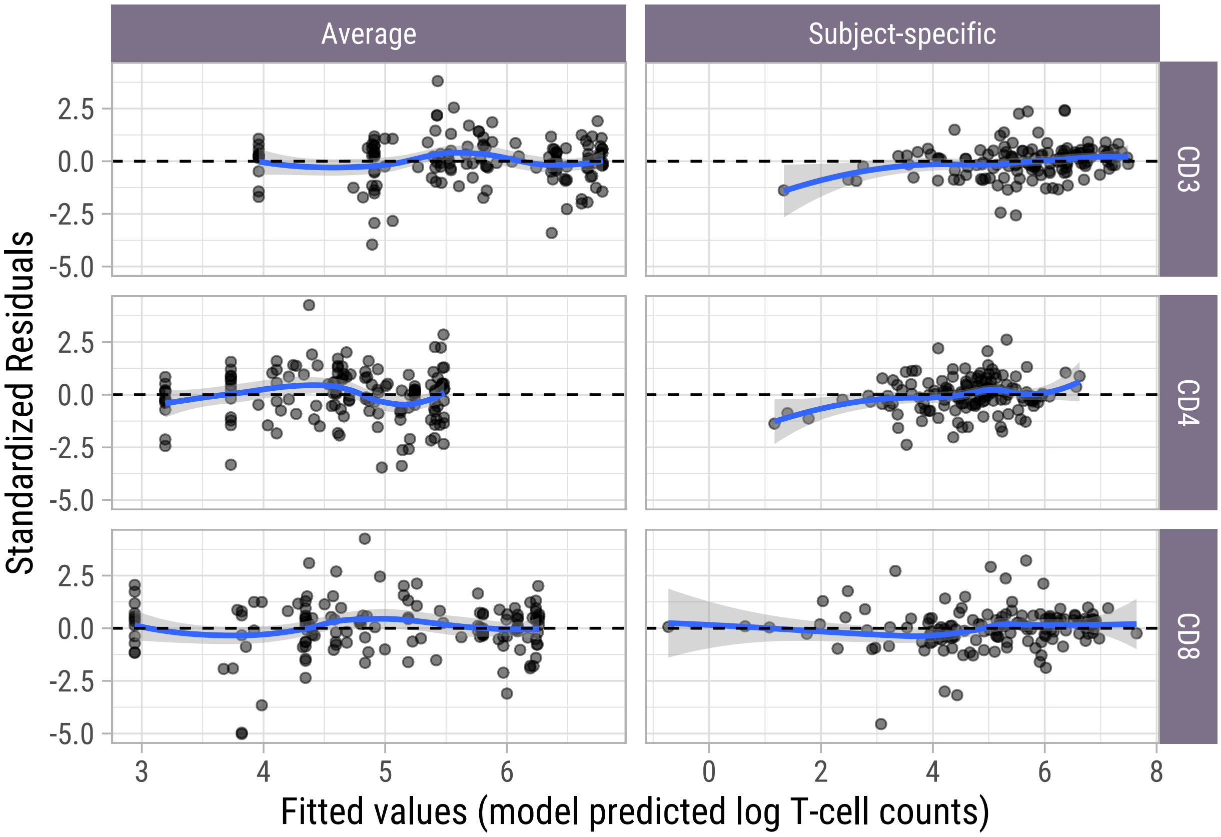

Supplement: Supplementary file 2 [file DataSheet_2.pdf]
